# tRNAs as a Driving Force of Genome Evolution in Yeast

**DOI:** 10.3389/fmicb.2021.634004

**Published:** 2021-03-11

**Authors:** Ana Rita Guimarães, Inês Correia, Inês Sousa, Carla Oliveira, Gabriela Moura, Ana Rita Bezerra, Manuel A. S. Santos

**Affiliations:** Department of Medical Sciences, Institute of Biomedicine – iBiMED, University of Aveiro, Aveiro, Portugal

**Keywords:** tRNA, yeast, chromatin structure, chromosome architecture, genome evolution, genomic instability

## Abstract

Transfer RNAs (tRNAs) are widely known for their roles in the decoding of the linear mRNA information into amino acid sequences of proteins. They are also multifunctional platforms in the translation process and have other roles beyond translation, including sensing amino acid abundance, interacting with the general stress response machinery, and modulating cellular adaptation, survival, and death. In this mini-review, we focus on the emerging role of tRNA genes in the organization and modification of the genomic architecture of yeast and the role of tRNA misexpression and decoding infidelity in genome stability, evolution, and adaption. We discuss published work showing how quickly tRNA genes can mutate to meet novel translational demands, how tRNAs speed up genome evolution, and how tRNA genes can be sites of genomic instability. We highlight recent works showing that loss of tRNA decoding fidelity and small alterations in tRNA expression have unexpected and profound impacts on genome stability. By dissecting these recent evidence, we hope to lay the groundwork that prompts future investigations on the mechanistic interplay between tRNAs and genome modification that likely triggers genome evolution.

## Introduction

Transfer RNAs (tRNAs) are short non-coding RNAs, approximately 70 to 100 bases long, that play essential roles in translation by linking mRNA codons to their corresponding amino acids, following a set of decoding rules established by the genetic code. They do so by base pairing their anticodon triplets with mRNA codon triplets in the ribosome decoding center and transferring the amino acid attached to its 3′-end in the ribosomal peptidyl transferase center (Phizicky and Hopper, 2010). This is a critical cellular process that requires tight control of tRNA gene expression, tRNA maturation, tRNA charging, and turnover (Chan and Lowe, 2016). In actively dividing yeast cells, tRNAs represent approximately 15% of total RNA (Warner, 1999), indicating that their genes (tDNAs) are highly transcribed. In general, tDNAs are nucleosome free and are flanked by strongly positioned nucleosomes (Yuan et al., 2005; Cole et al., 2012). Their transcription is mediated by RNA polymerase III (Pol III) upon recruitment to the promoter by the transcription factors TFIIIC and TFIIIB. TFIIIC binds to the internal A-box and B-box promoter elements and helps recruit the multi-subunit factor TFIIIB to AT-rich sequences upstream of the transcription start site, forming a highly stable TFIIIB–DNA complex that participates in multiple rounds of Pol III recruitment and initiation (Schramm and Hernandez, 2002). In *Saccharomyces cerevisiae*, the cellular concentration of each tRNA is directly proportional to its gene copy number (Percudani et al., 1997). This is particularly important because translation efficiency is described as the degree to which the tRNA pool can accommodate the transcriptome, thus affecting protein production and accuracy (dos Reis et al., 2004). This interplay is fine-tuned by codon usage, which is under selective pressure and show variation across budding yeast species (LaBella et al., 2019). Yet tRNAs have other non-canonical roles in the biological theater beyond their role as adaptors in protein synthesis (reviewed in Raina and Ibba, 2014 and Su et al., 2020). For example, tDNAs have roles in chromatin organization and gene regulation and are sites for binding of numerous chromatin proteins, including the architectural structural maintenance of chromosomes (SMC) proteins, nuclear pore proteins, chromatin remodelers, and histone modifiers (D’Ambrosio et al., 2008; Su et al., 2020).

The 275 tDNAs present in the yeast *S. cerevisiae* genome are dispersed throughout the linear maps of the 16 chromosomes. Fluorescence *in situ* hybridization microscopy (FISH) showed that tDNAs cluster at the outer periphery of the nucleolus in a microtubule-dependent manner and or adjacent to centromeres (Thompson et al., 2003). This happens with the assistance of condensing complexes bound at each tDNA gene locus (Haeusler et al., 2008) and requires substantial rearrangements of the genome topology. Whether individual tDNA associations play a role in genome organization is still poorly understood. We review below recent works on how tDNAs and related Pol III promoter elements function as boundary elements that limit chromatin domains (Donze and Kamakaka, 2001), how they work as barriers to DNA replication fork progression, and how they contribute to the formation of genomic fragile sites (Pryce et al., 2009). Beyond their role in the three-dimensional and functional organization of the genome, this review also describes how changes in the tRNA pool can drive genome evolution in fungi.

## Roles of tRNA Genes in Chromatin Remodeling and Genome Organization

The three-dimensional organization of the genome can promote long-range genomic rearrangements between interacting loci whose associated chromatin and transcriptional states can be selected through evolution (Bagadia et al., 2016). In yeast, tRNA genes have been implicated in the spatial organization of the genome by acting as barrier elements and by regulating chromatin structure (Donze and Kamakaka, 2001; Noma et al., 2006; Simms et al., 2008; Iwasaki et al., 2010; Hamdani et al., 2019). Evidence that tDNAs can hamper silenced chromatin domains from invading active domains was first obtained in *S. cerevisiae* (Donze and Kamakaka, 2001; Simms et al., 2004), where the deletion of a Thr-tRNA_*AGU*_ gene at the transcriptionally silent HMR mating-type locus resulted in the spread of silencing and consequent repression of the *GIT1* gene on chromosome III (Donze and Kamakaka, 2001). A Gln-tRNA_*UUG*_ gene has also been shown to block silencing at the *S. cerevisiae* rDNA locus (RDN1) (Biswas et al., 2009). Insulator activity was similarly shown in *Schizosaccharomyces pombe*, where deletion of a centromeric Ala-tRNA gene led to the spread of pericentromeric heterochromatin and gene silencing (Scott et al., 2006). The precise mechanisms by which tDNAs exert their barrier function remain largely unexplored; however, the assembly of the complete Pol III transcription apparatus does seem to be required for barrier function (Donze and Kamakaka, 2001; Scott et al., 2006; Biswas et al., 2009). Mutations in internal Thr-tRNA promoter elements, A-box or B-box, at the HMR locus led to deficiencies of TFIIIC and TFIIIB assembly, resulting in the loss of barrier function in *S. cerevisiae* (Donze and Kamakaka, 2001). Furthermore, yeast cohesin complex mutants (Δ*smc1* and Δ*smc3*) have impaired tDNA-mediated insulator function (Donze et al., 1999).

A study by Duan et al. (2010) mapped *cis-* and *trans-*interactions across the entire genome in *S. cerevisiae* and showed that physical interactions among tDNAs are significantly enriched and that they largely co-localize into clusters associated with the nucleolus or centromeres (Duan et al., 2010). Other studies, using DNA FISH, also showed that some tRNA genes cluster together near centromeres (Thompson et al., 2003). Furthermore, microscopic observations and genome-wide mapping of physical interactions show the co-localization of TFIIIC, cohesins, and other structural proteins at tDNA physical domain borders, suggesting that these insulators are critical players in chromosome folding and organization in the yeast nucleus. Recently, Hamdani et al. (2019) devised a strategy to tackle this topic. They eliminated the internal promoter elements (A-box and B-box) of two tDNAs on the left arm and eight tDNAs on the right arm of chromosome III in *S. cerevisiae* to generate a “tDNA-less” chromosome where binding of transcription factors TFIIIC and TFIIIB and chromatin proteins was abrogated. This allowed the detailed characterization of chromatin packaging, folding, and nuclear dynamics of chromosome III. Using various approaches, such as MNase-seq, ChIP-seq, RNA-seq, and fluorescence microscopy co-localization analysis, authors showed that (1) tDNA loss affects chromatin structure by disrupting the precise nucleosome positioning outside tDNAs; (2) tDNAs are essential to recruitment of cohesins and condensins; and (3) tDNAs influence centromere clustering, which in turn affects nuclear architecture. Lastly, as in previous studies (Donze and Kamakaka, 2001; Simms et al., 2004; Biswas et al., 2009), loss of tDNAs alters the long-range interactions of the silenced HML and HMR loci of chromosome III, leading to alterations in gene silencing (Hamdani et al., 2019).

The discovery of tDNA insulator function in yeast along with the recent advances in uncovering their involvement in the functional and spatial organization of the genome is particularly relevant because they provide a framework for future studies in this field. Furthermore, tDNA insulator functions seem to be conserved from yeast to humans (Raab et al., 2012), and their activities appear to be associated with a significant number of protein complexes whose actions and regulation remain to be determined.

## tRNA Genes, R-Loops, and Transposable Elements

tDNAs are often located near naturally occurring genomic fragile sites, and genome-wide studies in *S. cerevisiae* have detected R-loops at tRNA genes, along with other Pol III transcribed genes (Chan et al., 2014; El Hage et al., 2014; Wahba et al., 2016; Yeung and Smith, 2020). The replication machinery naturally slows down at tDNAs, and DNA helicases must take action to promote the progression of the replication fork (Ivessa et al., 2003). However, when the direction of DNA replication conflicts with the direction of the tDNA transcription, it leads to replication-fork pausing (Osmundson et al., 2017; Yeung and Smith, 2020). Head-on replication-fork pausing promotes DNA damage by R-loop formation (Tran et al., 2017). R-loops are stable DNA:RNA hybrid structures with an unpaired DNA strand that naturally blocks replication but can also generate genomic instability (Santos-Pereira and Aguilera, 2015). If left unresolved, R-loops can create replication–transcription conflicts and lead to double-strand breaks which potentially increase DNA recombination (Hegazy et al., 2020). Tran et al. (2017) showed that tDNAs represent sites of double-strand breaks and of increased recombination events in a series of helicase mutants. Moreover, this phenomenon is also intimately connected with high expression levels of tRNAs and with the fact that tDNAs are usually associated with the pre-initiation complex, *i.e.*, at a ready transcription state, which is a stable multiprotein complex consisting of a constant passage barrier for helicases (Arimbasseri et al., 2014).

Comparative genomics of 11 evolutionary-related yeast species showed a prevalence of tRNA genes at DNA breakpoints, which have also been linked to sites of genomic rearrangement (Gordon et al., 2009). One of the aspects that could underlie this observation is the preferential integration of transposable elements (TEs) at the proximity of tDNAs (Hani and Feldmann, 1998). TEs are mobile self-replicating elements that can integrate themselves in new genomic sites, being a potential source of mutations. There is an underlying assumption that TE insertions are deleterious, and indeed, they are a potential threat to genome integrity. *S. cerevisiae* has five families of TEs classified as long terminal repeat (LTR) retrotransposons, *Ty1* to *Ty5*. The most abundant and active ones are the Ty1 and Ty2, which, apart from their ORFs, share a high sequence similarity between their LTR sequences (Carr et al., 2012). These long and near-identical sequences scattered in the genome are prone to recombination, particularly ectopic recombination, which allows for an array of rearrangements like deletions, duplications, inversions, and translocations (Mieczkowski et al., 2006). It is therefore important that a tight control of retrotransposons’ expression is maintained. Ty1 mobility is regulated by a retrograde mechanism where Ty1 self-encoded elements, like p22, inhibits Ty1’s mobility when an elevated number of copies are present (Saha et al., 2015). This ability in *S. cerevisiae* was acquired by horizontal transfer from *Saccharomyces paradoxus* (Czaja et al., 2020). Nevertheless, comparative studies have shown that a large percentage of TEs are fixed in the genome (Bensasson, 2011), although there is also evidence for recent *Ty* insertions at a high rate (Carr et al., 2012), which can be seen as a source of genomic diversity and evolution. Increased retromobility has been observed upon exposure to several stress conditions like UV light (Bradshaw and McEntee, 1989) and adenine starvation (Todeschini et al., 2005). Furthermore, in physiological conditions like aging, retromobility has been observed in several species (Dennis et al., 2012; De Cecco et al., 2013; Li et al., 2013). In yeast, Maxwell et al. (2011) reported that during chronological aging, there is an association between Ty1 mobility and the observed genomic instability, particularly in loss of heterozygosity (LOH) events. Important adaptative roles for transposons have also been reported in experimentally evolved yeast. Dunham et al. (2002) studied the recombination events in evolved strains under glucose limitation and found that almost all detected rearrangements could be traced to ectopic rearrangement between transposons, transposon fragments, or tRNAs. In an experimental evolution study of cells expressing a mutant Ser-tRNA (see below), there were large chromosomal rearrangements mediated by homologous recombination between transposons (Kalapis et al., 2015). In a large timescale, phylogenetic studies on genome evolution identified tRNAs and transposons at genome breakpoints and rearrangement sites (Fischer et al., 2000; Kellis et al., 2003; Gordon et al., 2009). Interestingly, in a comparative evolutionary study between *S. cerevisiae* and its related species *S. paradoxus*, *S. mikatae*, and *S. uvarum*, all the inversions identified were flanked by tDNAs in an opposite transcriptional orientation (Kellis et al., 2003). Thus, it is apparent that tDNAs, or their flaking regions, play an important role in genome innovation and evolution. Although it was not acknowledged in any study (at least to our understanding), it is possible that tDNAs (and their vicinity) represent “silent hotspots” for recombination that, when a particular condition is prolonged, become sites for “rapid” adaptive genomic alterations.

## Genomic Changes Associated With tRNA Misexpression

Upon environmental challenges, the organism quickly needs a particular set of defenses to survive. There is a body of evidence on how transcription changes in response to several stresses in yeast (Gasch et al., 2000; Gutin et al., 2019). It is, therefore, reasonable to assume that tRNA expression and abundance must also be tuned to follow these changes. Indeed, the tRNA pool dynamically changes to facilitate selective and faster translation of stress-related transcripts (Torrent et al., 2018). The tRNA pool is composed of various tRNA isoacceptor families, each encoded by tDNAs with different copy numbers. tRNA gene families with more copies of the same tDNA decode more frequently used codons, while tRNAs with one gene copy decode rarely used codons, which correlates with the codon usage of protein genes. This establishes the adequate balance between tRNA availability and the usage of its corresponding codon (Percudani et al., 1997). Curiously, not all copies of the same tRNA species contribute equally to the tRNA pool, and the loss of a particular copy can have different physiological consequences (Bloom-Ackermann et al., 2014). Thus, the multiplicity of copies enables higher expression of tRNAs in high translational demand and enables the tRNA pool to be dynamic enough to allow the dispensability of a particular copy to further expand the tRNA repertoire. This concept was explored by Yona et al. (2013) in a yeast strain with a deletion in the single-copy tDNA *tR(CCU)J* (Bloom-Ackermann et al., 2014), thus eliminating the only cognate tRNA for the AGG codon. Experimental evolution revealed that 200 generations were sufficient for cells to overcome the translational defect. Translational equilibrium was restored by mutating the anticodon of one of the 11 copies of Arg-tRNA_*UCU*_ from UCU to CCU, without affecting cellular fitness, highlighting how the plasticity of the tRNA pool can overcome translational challenges in changing environments.

Maintaining the proteome’s good health is of extreme importance, but several bacterial and fungal species are able to decrease translation fidelity during stress to functionally diversify the proteome, a phenomenon called adaptive translation (Pan, 2013). Although alterations in the identity of a sense codon are a rare phenomenon, several budding yeasts reassigned the CUG codon to serine (Santos and Tuite, 1995) and to alanine (Muhlhausen et al., 2016; Riley et al., 2016). The CUG reassignments occurred independently during evolution and involved different tRNA genes that convergently mutated anticodons to CAG (Krassowski et al., 2018). *Candida albicans* is the most studied example of adaptive translation, where the identity of the CUG codon was altered to serine but residual leucine identity was still maintained. This results in an ambiguous CUG codon that is translated 97% of the times as serine and 3% as leucine, in standard growth conditions (Gomes et al., 2007). This is accomplished by a single Ser-tRNA_*CAG*_ with identity elements for both seryl- and leucyl-tRNA synthetases (Suzuki et al., 1997). However, alteration of the levels of the CUG-decoding tRNA is surprisingly adaptative. Bezerra et al. (2013) engineered a set of *C. albicans* strains with different combinations of tDNA_*CAG*_ copy number, where one, two, or both copies of the endogenous Ser-tRNA_*CAG*_ genes were deleted and one or two copies of the *S. cerevisiae* Leu-tRNA_*CAG*_ genes were inserted, thus shifting the ratio of leucine/serine incorporated in the proteome. Strains tolerated increasing Leu incorporation and displayed unexpected phenotypic variability, with highly variable colony and cell morphologies, and increased tolerance to fluconazole and itraconazole. Interestingly, altering the copy number of the CUG decoding tDNAs leads to the rapid accumulation of unique single-nucleotide polymorphisms (SNPs) and LOH events. Strains with higher deregulation of the tRNA pool, and therefore higher levels of Leu incorporation at CUG sites, showed higher number of SNPs, indicating the potential mutagenic effect of tRNA codon misreading. Of note was the fact that strains with the most extreme alterations in the tRNA pool (*i.e*., with highest level of Leu incorporation) presented a near-complete LOH on chromosome V. A set of genes related with stress response, antifungal drug resistance, filamentous growth, and pathogenesis is located in this chromosome, showing that these alterations are not random and have an adaptative role (Bezerra et al., 2013). One could hypothesize that the observed genomic alterations triggered by tRNA misexpression are associated with the peculiar features of the *C. albicans* biology and its highly plastic genome (Selmecki et al., 2010). However, a similar phenotype was also uncovered in *S. cerevisiae*. Kalapis et al. (2015) experimentally evolved a yeast strain engineered with a mutant Ser-tRNA_*CAG*_ that misincorporates serine at CUG codons. Although this insertion was highly detrimental for fitness, cells were able to adapt to their new condition after 250 generations to their new condition. Genome sequencing showed that tolerance and adaptation to translational stress were achieved by large genomic rearrangements. These repeatedly involved a partial deletion of 127 kb at chromosome V, enriched with genes involved in deubiquitination processes, and a duplication of 540 kb in chromosome IV, enriched with genes involved in glucose uptake. Together, these allowed cells to adapt to imbalances in the tRNA pool that culminate in CUG mistranslation by accelerated protein turnover and a high rate of glucose internalization (Kalapis et al., 2015). In other words, alterations in the tRNA pool and mRNA decoding accuracy destabilize the proteome in a dynamic way that reciprocates to the genome and produce important adaptive genome instabilities.

## Conclusion and Future Perspectives

**FIGURE 1:**
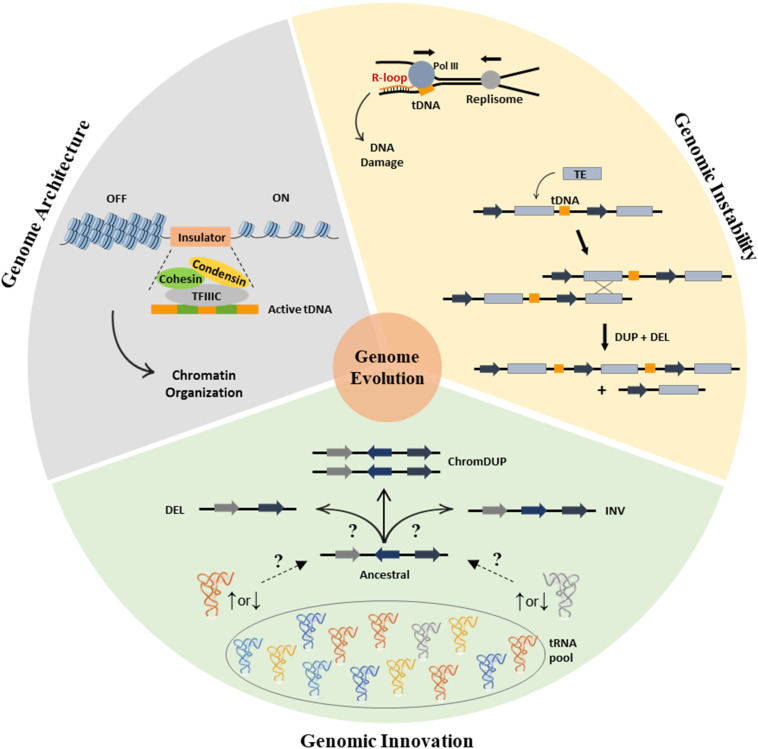
The involvement of tRNAs in genome organization and evolution. (Gray section) Role of tDNAs in genome architecture. TFIII recognizes the tDNA promoters (green boxes) enabling the assembly of the complete Pol III transcription apparatus and the recruitment of cohesin and condensin. Recruitment of the latter is crucial as it blocks the spread of heterochromatin (silenced state, OFF) into the active euchromatin (ON). (Yellow section) Contribution of tDNAs for genomic instability. tRNA genes are known sites for R-loop formation, which can be precursors of genomic instability, particularly when the directions of genome replication and of tRNA transcription collide (top). TEs integrate into the genome preferentially upstream of tDNAs. TEs are prone sites for ectopic recombination, like the one depicted, where recombination between sister chromatids results in deletions and duplications of the sequences located within the TEs (bottom). (Green section) The elusive connection between alterations in the tRNA pool and the genome instability. Alterations of tRNA’s abundance level destabilize the proteome, leading to adaptive genome instability and mutations through poorly understood mechanisms (represented by “?”).

We highlighted the non-canonical function of tRNAs and tDNAs as drivers of genome evolution (Figure 1) and summarized how tDNA can play a role in the three-dimensional and functional organization of the genome and potentiate genome rearrangements events. Additionally, the discovery that alterations in the yeast tRNA pool generate genome instability associated with phenotypic variation of high adaptation potential adds a new dimension to the study of tRNA-driven genome evolution. Precisely how these mechanisms operate remains to be determined, but future work should elucidate how the tRNA pool provides evolutionary plasticity in environmental changing conditions. It is of high biological importance to understand the complex relationship between the tRNA pool and the genome, since the produced genomic instabilities may be relevant to human diseases, including cancer where extensive tRNA pool alterations and aneuploidies have been observed.

## Author Contributions

AG and AB: conceptualization, literature study, writing, and editing of the manuscript. MS: editing and revision of the manuscript. All authors listed have made a substantial, direct and intellectual contributions to the work, and approved it for publication.

## Conflict of Interest

The authors declare that the research was conducted in the absence of any commercial or financial relationships that could be construed as a potential conflict of interest.

## References

[B1] Arimbasseri A. G. Rijal K. Maraia R. J. (2014). Comparative overview of RNA polymerase II and III transcription cycles, with focus on RNA polymerase III termination and reinitiation. *Transcription* 5:e27639. 10.4161/trns.27369 25764110 PMC4214234

[B2] Bagadia M. Singh A. Singh Sandhu K. (2016). Three dimensional organization of genome might have guided the dynamics of gene order evolution in eukaryotes. *Genome Biol. Evol*. 8 946–954. 10.1093/gbe/evw050 26957031 PMC4824123

[B3] Bensasson D. (2011). Evidence for a high mutation rate at rapidly evolving yeast centromeres. *BMC Evol. Biol*. 11:211. 10.1186/1471-2148-11-211 21767380 PMC3155921

[B4] Bezerra A. R. Simoes J. Lee W. Rung J. Weil T. Gut I. G. (2013). Reversion of a fungal genetic code alteration links proteome instability with genomic and phenotypic diversification. *Proc. Natl. Acad. Sci. U.S.A*. 110 11079–11084. 10.1073/pnas.1302094110 23776239 PMC3704024

[B5] Biswas M. Maqani N. Rai R. Kumaran S. P. Iyer K. R. Sendinc E. (2009). Limiting the extent of the RDN1 heterochromatin domain by a silencing barrier and Sir2 protein levels in *Saccharomyces cerevisiae*. *Mol. Cell. Biol*. 29 2889–2898. 10.1128/MCB.00728-08 19289503 PMC2682026

[B6] Bloom-Ackermann Z. Navon S. Gingold H. Towers R. Pilpel Y. Dahan O. (2014). A comprehensive tRNA deletion library unravels the genetic architecture of the tRNA pool. *PLoS Genet*. 10:e1004084. 10.1371/journal.pgen.1004084 24453985 PMC3894157

[B7] Bradshaw V. A. McEntee K. (1989). DNA damage activates transcription and transposition of yeast Ty retrotransposons. *Mol. Gen. Genet*. 218 465–474. 10.1007/BF00332411 2555668

[B8] Carr M. Bensasson D. Bergman C. M. (2012). Evolutionary genomics of transposable elements in *Saccharomyces cerevisiae*. *PLoS One* 7:e50978. 10.1371/journal.pone.0050978 23226439 PMC3511429

[B9] Chan P. P. Lowe T. M. (2016). GtRNAdb 2.0: an expanded database of transfer RNA genes identified in complete and draft genomes. *Nucleic Acids Res*. 44 D184–D189. 10.1093/nar/gkv1309 26673694 PMC4702915

[B10] Chan Y. A. Aristizabal M. J. Lu P. Y. Luo Z. Hamza A. Kobor M. S. (2014). Genome-wide profiling of yeast DNA:RNA hybrid prone sites with DRIP-chip. *PLoS Genet*. 10:e1004288. 10.1371/journal.pgen.1004288 24743342 PMC3990523

[B11] Cole H. A. Howard B. H. Clark D. J. (2012). Genome-wide mapping of nucleosomes in yeast using paired-end sequencing. *Methods Enzymol*. 513 145–168. 10.1016/B978-0-12-391938-0.00006-9 22929768

[B12] Czaja W. Bensasson D. Ahn H. W. Garfinkel D. J. Bergman C. M. (2020). Evolution of Ty1 copy number control in yeast by horizontal transfer and recombination. *PLoS Genet*. 16:e1008632. 10.1371/journal.pgen.1008632 32084126 PMC7055915

[B13] D’Ambrosio C. Schmidt C. K. Katou Y. Kelly G. Itoh T. Shirahige K. (2008). Identification of cis-acting sites for condensin loading onto budding yeast chromosomes. *Genes Dev*. 22 2215–2227. 10.1101/gad.1675708 18708580 PMC2518811

[B14] De Cecco M. Criscione S. W. Peterson A. L. Neretti N. Sedivy J. M. Kreiling J. A. (2013). Transposable elements become active and mobile in the genomes of aging mammalian somatic tissues. *Aging* 5 867–883. 10.18632/aging.100621 24323947 PMC3883704

[B15] Dennis S. Sheth U. Feldman J. L. English K. A. Priess J. R. (2012). C. elegans germ cells show temperature and age-dependent expression of Cer1, a Gypsy/Ty3-related retrotransposon. *PLoS Pathog*. 8:e1002591. 10.1371/journal.ppat.1002591 22479180 PMC3315495

[B16] Donze D. Adams C. R. Rine J. Kamakaka R. T. (1999). The boundaries of the silenced HMR domain in *Saccharomyces cerevisiae*. *Genes Dev*. 13 698–708. 10.1101/gad.13.6.698 10090726 PMC316548

[B17] Donze D. Kamakaka R. T. (2001). RNA polymerase III and RNA polymerase II promoter complexes are heterochromatin barriers in *Saccharomyces cerevisiae*. *EMBO J*. 20 520–531. 10.1093/emboj/20.3.520 11157758 PMC133458

[B18] dos Reis M. Savva R. Wernisch L. (2004). Solving the riddle of codon usage preferences: a test for translational selection. *Nucleic Acids Res*. 32 5036–5044. 10.1093/nar/gkh834 15448185 PMC521650

[B19] Duan Z. Andronescu M. Schutz K. McIlwain S. Kim Y. J. Lee C. (2010). A three-dimensional model of the yeast genome. *Nature* 465 363–367. 10.1038/nature08973 20436457 PMC2874121

[B20] Dunham M. J. Badrane H. Ferea T. Adams J. Brown P. O. Rosenzweig F. (2002). Characteristic genome rearrangements in experimental evolution of *Saccharomyces cerevisiae*. *Proc. Natl. Acad. Sci. U.S.A*. 99 16144–16149. 10.1073/pnas.242624799 12446845 PMC138579

[B21] El Hage A. Webb S. Kerr A. Tollervey D. (2014). Genome-wide distribution of RNA-DNA hybrids identifies RNase H targets in tRNA genes, retrotransposons and mitochondria. *PLoS Genet*. 10:e1004716. 10.1371/journal.pgen.1004716 25357144 PMC4214602

[B22] Fischer G. James S. A. Roberts I. N. Oliver S. G. Louis E. J. (2000). Chromosomal evolution in *Saccharomyces*. *Nature* 405 451–454. 10.1038/35013058 10839539

[B23] Gasch A. P. Spellman P. T. Kao C. M. Carmel-Harel O. Eisen M. B. Storz G. (2000). Genomic expression programs in the response of yeast cells to environmental changes. *Mol. Biol. Cell* 11 4241–4257. 10.1091/mbc.11.12.4241 11102521 PMC15070

[B24] Gomes A. C. Miranda I. Silva R. M. Moura G. R. Thomas B. Akoulitchev A. (2007). A genetic code alteration generates a proteome of high diversity in the human pathogen *Candida albicans*. *Genome Biol*. 8:R206. 10.1186/gb-2007-8-10-r206 17916231 PMC2246281

[B25] Gordon J. L. Byrne K. P. Wolfe K. H. (2009). Additions, losses, and rearrangements on the evolutionary route from a reconstructed ancestor to the modern *Saccharomyces cerevisiae* genome. *PLoS Genet*. 5:e1000485. 10.1371/journal.pgen.1000485 19436716 PMC2675101

[B26] Gutin J. Joseph-Strauss D. Sadeh A. Shalom E. Friedman N. (2019). Genetic screen of the yeast environmental stress response dynamics uncovers distinct regulatory phases. *Mol. Syst. Biol*. 15:e8939. 10.15252/msb.20198939 31464369 PMC6711295

[B27] Haeusler R. A. Pratt-Hyatt M. Good P. D. Gipson T. A. Engelke D. R. (2008). Clustering of yeast tRNA genes is mediated by specific association of condensin with tRNA gene transcription complexes. *Genes Dev*. 22 2204–2214. 10.1101/gad.1675908 18708579 PMC2518813

[B28] Hamdani O. Dhillon N. Hsieh T. S. Fujita T. Ocampo J. Kirkland J. G. (2019). tRNA genes affect chromosome structure and function via local effects. *Mol. Cell. Biol*. 39 e00432–18. 10.1128/MCB.00432-18 30718362 PMC6447413

[B29] Hani J. Feldmann H. (1998). tRNA genes and retroelements in the yeast genome. *Nucleic Acids Res*. 26 689–696. 10.1093/nar/26.3.689 9443958 PMC147333

[B30] Hegazy Y. A. Fernando C. M. Tran E. J. (2020). The balancing act of R-loop biology: the good, the bad, and the ugly. *J. Biol. Chem*. 295 905–913. 10.1074/jbc.REV119.011353 31843970 PMC6983857

[B31] Ivessa A. S. Lenzmeier B. A. Bessler J. B. Goudsouzian L. K. Schnakenberg S. L. Zakian V. A. (2003). The *Saccharomyces cerevisiae* helicase Rrm3p facilitates replication past nonhistone protein-DNA complexes. *Mol. Cell* 12 1525–1536. 10.1016/s1097-2765(03)00456-8 14690605

[B32] Iwasaki O. Tanaka A. Tanizawa H. Grewal S. I. Noma K. (2010). Centromeric localization of dispersed Pol III genes in fission yeast. *Mol. Biol. Cell* 21 254–265. 10.1091/mbc.E09-09-0790 19910488 PMC2808234

[B33] Kalapis D. Bezerra A. R. Farkas Z. Horvath P. Bodi Z. Daraba A. (2015). Evolution of robustness to protein mistranslation by accelerated protein turnover. *PLoS Biol*. 13:e1002291. 10.1371/journal.pbio.1002291 26544557 PMC4636289

[B34] Kellis M. Patterson N. Endrizzi M. Birren B. Lander E. S. (2003). Sequencing and comparison of yeast species to identify genes and regulatory elements. *Nature* 423 241–254. 10.1038/nature01644 12748633

[B35] Krassowski T. Coughlan A. Y. Shen X. X. Zhou X. Kominek J. Opulente D. A. (2018). Evolutionary instability of CUG-Leu in the genetic code of budding yeasts. *Nat. Commun*. 9:1887. 10.1038/s41467-018-04374-7 29760453 PMC5951914

[B36] LaBella A. L. Opulente D. A. Steenwyk J. L. Hittinger C. T. Rokas A. (2019). Variation and selection on codon usage bias across an entire subphylum. *PLoS Genet*. 15:e1008304. 10.1371/journal.pgen.1008304 31365533 PMC6701816

[B37] Li W. Prazak L. Chatterjee N. Gruninger S. Krug L. Theodorou D. (2013). Activation of transposable elements during aging and neuronal decline in *Drosophila*. *Nat. Neurosci*. 16 529–531. 10.1038/nn.3368 23563579 PMC3821974

[B38] Maxwell P. H. Burhans W. C. Curcio M. J. (2011). Retrotransposition is associated with genome instability during chronological aging. *Proc. Natl. Acad. Sci. U.S.A*. 108 20376–20381. 10.1073/pnas.1100271108 22021441 PMC3251071

[B39] Mieczkowski P. A. Lemoine F. J. Petes T. D. (2006). Recombination between retrotransposons as a source of chromosome rearrangements in the yeast *Saccharomyces cerevisiae*. *DNA Repair* 5 1010–1020. 10.1016/j.dnarep.2006.05.027 16798113

[B40] Muhlhausen S. Findeisen P. Plessmann U. Urlaub H. Kollmar M. (2016). A novel nuclear genetic code alteration in yeasts and the evolution of codon reassignment in eukaryotes. *Genome. Res*. 26 945–955. 10.1101/gr.200931.115 27197221 PMC4937558

[B41] Noma K. Cam H. P. Maraia R. J. Grewal S. I. (2006). A role for TFIIIC transcription factor complex in genome organization. *Cell* 125 859–872. 10.1016/j.cell.2006.04.028 16751097

[B42] Osmundson J. S. Kumar J. Yeung R. Smith D. J. (2017). Pif1-family helicases cooperatively suppress widespread replication-fork arrest at tRNA genes. *Nat. Struct. Mol. Biol*. 24 162–170. 10.1038/nsmb.3342 27991904 PMC5296403

[B43] Pan T. (2013). Adaptive translation as a mechanism of stress response and adaptation. *Annu. Rev. Genet*. 47 121–137. 10.1146/annurev-genet-111212-133522 23988117 PMC4109725

[B44] Percudani R. Pavesi A. Ottonello S. (1997). Transfer RNA gene redundancy and translational selection in *Saccharomyces cerevisiae*. *J. Mol. Biol*. 268 322–330. 10.1006/jmbi.1997.0942 9159473

[B45] Phizicky E. M. Hopper A. K. (2010). tRNA biology charges to the front. *Genes Dev*. 24 1832–1860. 10.1101/gad.1956510 20810645 PMC2932967

[B46] Pryce D. W. Ramayah S. Jaendling A. McFarlane R. J. (2009). Recombination at DNA replication fork barriers is not universal and is differentially regulated by Swi1. *Proc. Natl. Acad. Sci. U.S.A*. 106 4770–4775. 10.1073/pnas.0807739106 19273851 PMC2660728

[B47] Raab J. R. Chiu J. Zhu J. Katzman S. Kurukuti S. Wade P. A. (2012). Human tRNA genes function as chromatin insulators. *EMBO J*. 31 330–350. 10.1038/emboj.2011.406 22085927 PMC3261562

[B48] Raina M. Ibba M. (2014). tRNAs as regulators of biological processes. *Front. Genet*. 5:171. 10.3389/fgene.2014.00171 24966867 PMC4052509

[B49] Riley R. Haridas S. Wolfe K. H. Lopes M. R. Hittinger C. T. Goker M. (2016). Comparative genomics of biotechnologically important yeasts. *Proc. Natl. Acad. Sci. U.S.A*. 113 9882–9887. 10.1073/pnas.1603941113 27535936 PMC5024638

[B50] Saha A. Mitchell J. A. Nishida Y. Hildreth J. E. Ariberre J. A. Gilbert W. V. (2015). A trans-dominant form of Gag restricts Ty1 retrotransposition and mediates copy number control. *J. Virol*. 89 3922–3938. 10.1128/JVI.03060-14 25609815 PMC4403431

[B51] Santos M. A. Tuite M. F. (1995). The CUG codon is decoded in vivo as serine and not leucine in *Candida albicans*. *Nucleic Acids Res*. 23 1481–1486. 10.1093/nar/23.9.1481 7784200 PMC306886

[B52] Santos-Pereira J. M. Aguilera A. (2015). R loops: new modulators of genome dynamics and function. *Nat. Rev. Genet*. 16 583–597. 10.1038/nrg3961 26370899

[B53] Schramm L. Hernandez N. (2002). Recruitment of RNA polymerase III to its target promoters. *Genes Dev*. 16 2593–2620. 10.1101/gad.1018902 12381659

[B54] Scott K. C. Merrett S. L. Willard H. F. (2006). A heterochromatin barrier partitions the fission yeast centromere into discrete chromatin domains. *Curr. Biol*. 16 119–129. 10.1016/j.cub.2005.11.065 16431364

[B55] Selmecki A. Forche A. Berman J. (2010). Genomic plasticity of the human fungal pathogen *Candida albicans*. *Eukaryot Cell* 9 991–1008. 10.1128/EC.00060-10 20495058 PMC2901674

[B56] Simms T. A. Dugas S. L. Gremillion J. C. Ibos M. E. Dandurand M. N. Toliver T. T. (2008). TFIIIC binding sites function as both heterochromatin barriers and chromatin insulators in *Saccharomyces cerevisiae*. *Eukaryot Cell* 7 2078–2086. 10.1128/EC.00128-08 18849469 PMC2593192

[B57] Simms T. A. Miller E. C. Buisson N. P. Jambunathan N. Donze D. (2004). The *Saccharomyces cerevisiae* TRT2 tRNAThr gene upstream of STE6 is a barrier to repression in MATalpha cells and exerts a potential tRNA position effect in MATa cells. *Nucleic Acids Res*. 32 5206–5213. 10.1093/nar/gkh858 15459290 PMC521669

[B58] Su Z. Wilson B. Kumar P. Dutta A. (2020). Noncanonical roles of tRNAs: tRNA fragments and beyond. *Annu. Rev. Genet*. 54 47–69. 10.1146/annurev-genet-022620-101840 32841070 PMC7686126

[B59] Suzuki T. Ueda T. Watanabe K. (1997). The ‘polysemous’ codon–a codon with multiple amino acid assignment caused by dual specificity of tRNA identity. *EMBO J*. 16 1122–1134. 10.1093/emboj/16.5.1122 9118950 PMC1169711

[B60] Thompson M. Haeusler R. A. Good P. D. Engelke D. R. (2003). Nucleolar clustering of dispersed tRNA genes. *Science* 302 1399–1401. 10.1126/science.1089814 14631041 PMC3783965

[B61] Todeschini A. L. Morillon A. Springer M. Lesage P. (2005). Severe adenine starvation activates Ty1 transcription and retrotransposition in *Saccharomyces cerevisiae*. *Mol. Cell. Biol*. 25 7459–7472. 10.1128/MCB.25.17.7459-7472.2005 16107695 PMC1190277

[B62] Torrent M. Chalancon G. de Groot N. S. Wuster A. Madan Babu M. (2018). Cells alter their tRNA abundance to selectively regulate protein synthesis during stress conditions. *Sci. Signal*. 11:eaat6409. 10.1126/scisignal.aat6409 30181241 PMC6130803

[B63] Tran P. L. T. Pohl T. J. Chen C. F. Chan A. Pott S. Zakian V. A. (2017). PIF1 family DNA helicases suppress R-loop mediated genome instability at tRNA genes. *Nat. Commun*. 8:15025. 10.1038/ncomms15025 28429714 PMC5413955

[B64] Wahba L. Costantino L. Tan F. J. Zimmer A. Koshland D. (2016). S1-DRIP-seq identifies high expression and polyA tracts as major contributors to R-loop formation. *Genes Dev*. 30 1327–1338. 10.1101/gad.280834.116 27298336 PMC4911931

[B65] Warner J. R. (1999). The economics of ribosome biosynthesis in yeast. *Trends Biochem. Sci*. 24 437–440. 10.1016/s0968-0004(99)01460-7 10542411

[B66] Yeung R. Smith D. J. (2020). Determinants of replication-fork pausing at tRNA genes in *Saccharomyces cerevisiae*. *Genetics* 214 825–838. 10.1534/genetics.120.303092 32071194 PMC7153945

[B67] Yona A. H. Bloom-Ackermann Z. Frumkin I. Hanson-Smith V. Charpak-Amikam Y. Feng Q. (2013). tRNA genes rapidly change in evolution to meet novel translational demands. *Elife* 2:e01339. 10.7554/eLife.01339 24363105 PMC3868979

[B68] Yuan G. C. Liu Y. J. Dion M. F. Slack M. D. Wu L. F. Altschuler S. J. (2005). Genome-scale identification of nucleosome positions in *S. cerevisiae*. *Science* 309 626–630. 10.1126/science.1112178 15961632

